# Day Surgery Program at West China Hospital: Exploring the Initial Experience

**DOI:** 10.7759/cureus.8961

**Published:** 2020-07-02

**Authors:** Lisha Jiang, Rebecca Houston, Chao Li, Javed Siddiqi, Qingxin Ma, Shanzun Wei, Hongsheng Ma

**Affiliations:** 1 Day Surgery Center, West China Hospital, Sichuan University, Chengdu, CHN; 2 Neurosurgery, Desert Regional Medical Center, Palm Springs, USA; 3 Neurosurgery, College of Osteopathic Medicine, Des Moines University, Des Moines, USA; 4 Neurosurgery, Riverside University Health System Medical Center, Moreno Valley, USA; 5 Neurosurgery, Arrowhead Regional Medical Center, Colton, USA; 6 Neurosurgery, California University of Science and Medicine, Colton, USA; 7 Psychology, West China Hospital, Sichuan University, Chengdu, CHN; 8 Urology, West China School of Medicine, Sichuan University, Chengdu, CHN

**Keywords:** day surgery, cost-effective, neurology, day care surgery

## Abstract

Healthcare facilities in China are facing increasing demands as the country has the fastest aging populations in the world. Day surgery can be utilized to address some of these demands. Benefits of day surgery include shortened hospital stay, decreased risk of hospital-associated infections, and increased cost efficiency. We present a retrospective study of eight years of day surgery data from West China Hospital, one of the largest hospitals in China, with an emphasis on an examination of the growth in day surgeries. We examined patterns of utilization of day surgery versus inpatient surgery (including types of surgeries performed in the Day Surgery Center and the ratio of day surgery versus elective surgery), as well as unplanned readmission and return to inpatient department rates, and a comparison of average costs and length of stay for day surgery versus hospital surgery.

Day surgery has a safe and cost-effective way to alleviate the soaring healthcare demands in West China. There is potential opportunity to further address the ever-increasing demands on the healthcare system in this area by performing more complex surgeries as day surgeries. This article presents an effective organizational protocol and proposes a reliable medical quality assurance system, which prioritizes safety of the growing day surgery program; furthermore, it describes the factors and lessons learned from the successful implementation of a day surgery system.

## Introduction

China offers many lessons regarding healthcare in a developing country. Healthcare facilities in China are facing increasing demands as the country has one of the fastest aging populations in the world [1]. Due to escalating medical demands, limited medical resources, and China’s record-setting pace of aging, the hospitals in China have been struggling to meet ever-increasing demands [2,3]. In response, China has been encouraging the development of general practitioners, reforming public hospitals, and strengthening primary care and social health insurance [4]. However, there remains opportunity to develop the system to serve the soaring medical demands in China. It is important to maximize the utilization of limited medical resources while still providing high-quality care for patients, and day surgery has the potential to achieve these goals [5].

Day surgery is also known as same-day surgery, ambulatory surgery, and outpatient surgery, with terms varying from country to country. The concept of day surgery dates back to 1899, as proposed by James H. Nicoll, who is known as the “father of day surgery” [6]. Day surgery is the admission of selected patients to a hospital or surgery center for a planned surgery or procedure with the expectation that the patient is to be discharged on the same day; it is a pathway of clinical management, not a specific kind of surgery or procedure. The definition of day surgery in West China Hospital (WCH) is a planned surgery that is performed, and the patient is discharged within 24 hours, excluding outpatient surgery, but the patient can stay overnight. It is similar to extended recovery of day surgery in the United Kingdom [7]. By comparison, with traditional surgery, the patient is generally admitted into a hospital for a stay expected to exceed 24 hours. The goals of day surgery are to reduce length of stay and to reduce cost without compromising the quality of care and patient satisfaction, making day surgery the preferred and dominant form of surgeries in some developed countries [8-10]. The first day surgery in China was performed in Hong Kong in 1990s and then followed by Shanghai, Sichuan, and Hubei [11,12].

## Materials and methods

Institutional Review Board approval

IRB approval was given by the Biomedical Research Ethics Committee of WCH, Sichuan University.

Data source

WCH, also as known as Hua’xi Hospital, is a medical center in southwest China, which has been ranked second in mainland China from 2009-2018 based on academic achievements and public reputation. It has 4,300 licensed beds. More than 5 million outpatient visits and 175,000 surgeries were recorded in 2018. It serves patients from all over the country [13]. WCH is among the first hospitals in mainland China with a standalone surgical center, which has 33 beds facilitating the performance of more than 150,000 surgeries over the past 10 years.

Data collection

The data were retrieved from the Hospital Information System from January 1, 2010, to December 31, 2018. A total of 140,738 cases were recorded. Relevant data points included admitting department, admission date, surgery date, surgery type, diagnoses, discharge date, and ICD-10 (International Classification of Diseases, 10th Revision) code. Diagnoses, procedures, or surgeries presented have been coded using the ICD-10, Clinical Modification (ICD-10-CM).

Methods

Establishment of Day Surgery Center

The successful implementation and operation of a day surgery service depend on coordination among clinical, administrative, and medical quality control staff and systems. Ever since the opening of the Day Surgery Center at WCH on October 28, 2009, administrative and clinical management practices have been consistently reviewed and revised as appropriate. Part of this protocol was the founding of a Day Surgery Management Committee of WCH, which includes an administrative supervisor and a department director, as well as multiple physicians and nurse representatives. Physician member selection is based on specialty and experience, as well as their enthusiasm and passion to promote and advance day surgery. WCH also established a Day Surgery Center patient service system, which provides a pre-operative assessment, surgery scheduling, healthcare education, coordination with pre-operative visit, assessment, and surgery scheduling. The department director and head nurse are in charge of the daily administration duties. Five physicians and 26 registered nurses were recruited for the Day Surgery Center for postoperative medical management.

Two Organizational Modes

**Figure 1:**
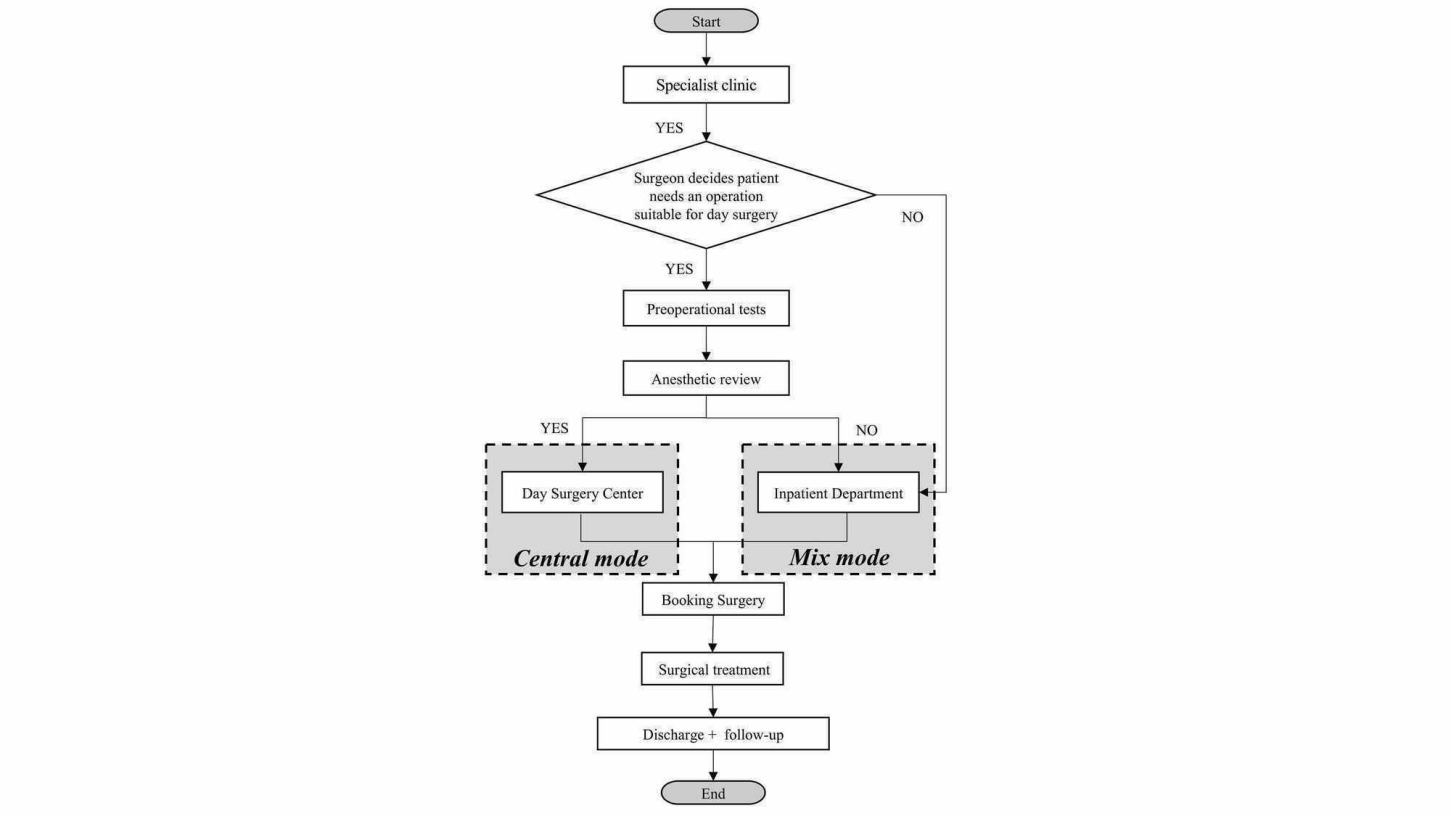
Admission and discharge procedures under two different organizational modes of day surgery in West China Hospital.

There exist two possible modes of managing admission and discharge flow for day surgery, as shown in Figure 1. Firstly, a central mode provides centralized pre- and postsurgical management for patients; Day Surgery Center is a case in point. Day Surgery Center of WCH is a hospital-based surgical center that contains five operating rooms, a post-anesthesia care unit, 33 beds, and two nurse stations. Surgery scheduling, surgery, short recovery, discharge, and telephone follow-up were done in Day Surgery Center. Secondly, a mixed mode is a centralized management and decentralized treatment way, which is less common at WCH but very popular in other hospitals for lower costs and easy management. It only requires a fraction of ward-beds in comparison to the inpatient surgical department for day surgery patients while the facilities and staff are the same. This mode is most advantageous to sites with a limited number of beds in their Day Surgery Center, which is why it is carried out in the Inpatient Department, mostly in urology, interventional cardiology, ophthalmology, and interventional neurology procedure.

Medical Quality Assurance System for Day Surgery

WCH established a new medical quality assurance system for day surgery, including patient selection, operation selection, surgeon qualification, nurse qualification, and team construction and management. How each dimension operates under this system is detailed as follows:

Patient selection: The patient must have adequate family and social support, especially for the first 24 hours after surgery. The patient must have access to transportation for 24 hours postoperatively either by a private vehicle with a driver other than the patient or by access to a 24-hour taxi service. They must reside at a less than two-hour driving distance from the hospital. Patient selection for day surgery is also based on their general health, age, and so on. The following basic requirements for patients have been proposed after the conclusion of practice:

1. The American Society of Anesthesiologists (ASA) scale: in general, only patients in class ASA I and II will be eligible for day surgery. Notably, there exists literature regarding ASA III patients undergoing day surgery, with low incidences of complications [14]. In WCH, day surgery can be arranged for ASA III patients if the comorbidities are stable for more than three months and are under constant monitoring.

2. Age: the literature demonstrates that day surgery has been successfully completed on patients of a wide range of ages [15,16]. The standard for day surgery eligibility in WCH varies by specialty. For example, pediatric surgery is performed on patients older than one year, adult laparoscopic cholecystectomy (LC) is performed on patients under 60 years of age, and the cutoff age for varicose vein and hernia repair is under 70 years.

3. Body mass index (BMI): being overweight or obese can increase the difficulty and incidence of postoperative complications. Hypertension, congestive heart failure, and snoring are the main postoperative complications associated with obese patients [17]. The British guidelines for day surgery stipulate that patients with a BMI of 35 kg/m2 can receive day surgery if fully maximized [18]. Due to the large differences in physique and diet between China and developed countries, day surgery is only performed on patients with a BMI of less than 35 kg/m2 at WCH [19].

4. Co-morbidities: patients with chronic diseases may still be considered for day surgery as long as their diseases are stable, known to the anesthesiologists and surgeons, and appropriately maximized for surgery by their medical team. Examples include hypertension, diabetes, and coronary artery atherosclerotic heart disease. However, end-stage diseases such as liver failure and kidney failure are excluded [20-22].

Surgery selection: A list of approved surgeries was developed by the Day Surgery Management Committee of WCH. Criteria are as follows: an estimated operation time less than two hours (so as to permit early ambulation and enhance rapid recovery), management of postoperative pain by prescription of oral analgesics only, and no special postoperative care back home.

Surgeon qualification: Surgeons must be senior attending physicians with experience over 10 years with the ability to operate independently and have completed a minimum number of cases, the amount of which is variable depending on the procedure. Example requirements include a minimum of 150 cases of hernia repair, LC, and vein stripping and ligation. Last but not least, these doctors must be enthusiastic and committed to the promotion and development of day surgery.

Nurse qualification: Nurses must have a minimum of 10 years of pre- and postsurgical care experience, strong communication skills, comprehensive knowledge of different specialties, and knowledge of hospital regulations, national policies, and medical insurance reimbursement procedures.

WCH follows clinical pathway protocol to optimize both clinical and non-clinical activities, including the financial and resource utilization components of care. By standardization of medical tests, orders, and treatment, clinical care safety and efficiency are maximized. Day Surgery Center has 5 physicians and 26 registered nurses assigned to either the pre-operative care team, peri-operative care team, or postoperative care team. The pre-operative care team consists of three senior nurses in the outpatient department. Their duties include double-checking the pre-operative tests and the management of surgery scheduling, health education, and inquiries. Five physicians are part of the peri-operative and postoperative care teams. They are responsible for reassessing surgical patients before surgery, medical record quality control, and taking care of patients postoperation. There are also four senior registered nurses in the postoperative care team. Their duties include follow-up and health education. Their primary focus is on postoperative complications that patients may experience at home. Discharged patients may reach the postoperative care team through telephone, a mobile application, or social media 24 hours a day, seven days a week for support managing any postoperative complications they may have, such as pain, nausea, and vomiting, or to make an appointment.

Growth of Day Surgeries

The number of day surgeries at WCH Day Surgery Center has increased steadily since its establishment. WCH began performing day surgeries on January 1, 2019. From that date through November 30, 2019, 36 cerebral angiography cases were performed, with no postoperative complications. The cancellation rate of booked procedures, unplanned return, and readmission rate remained zero. Additional procedures will be introduced as appropriate, such as performance of percutaneous micro-balloon decompression for the treatment of trigeminal neuralgia.

## Results

**Figure 2:**
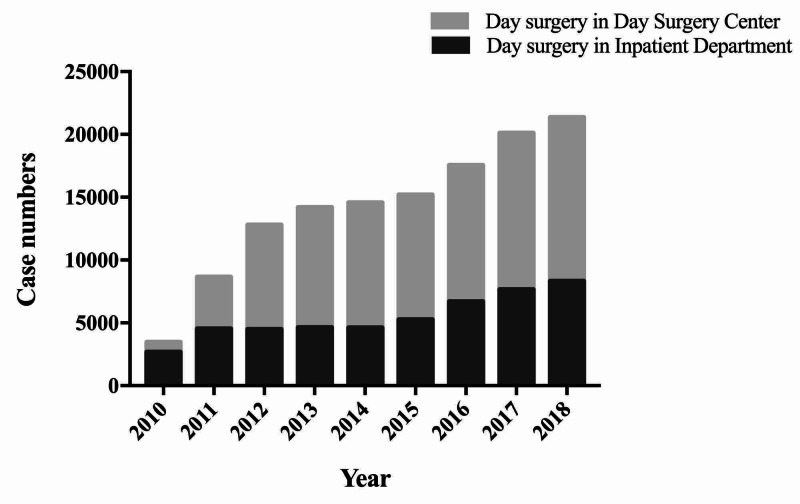
The total number of day surgeries performed at the WCH Day Surgery Center and WCH Inpatient Departments from 2010 to 2018. WCH, West China Hospital

**Figure 3:**
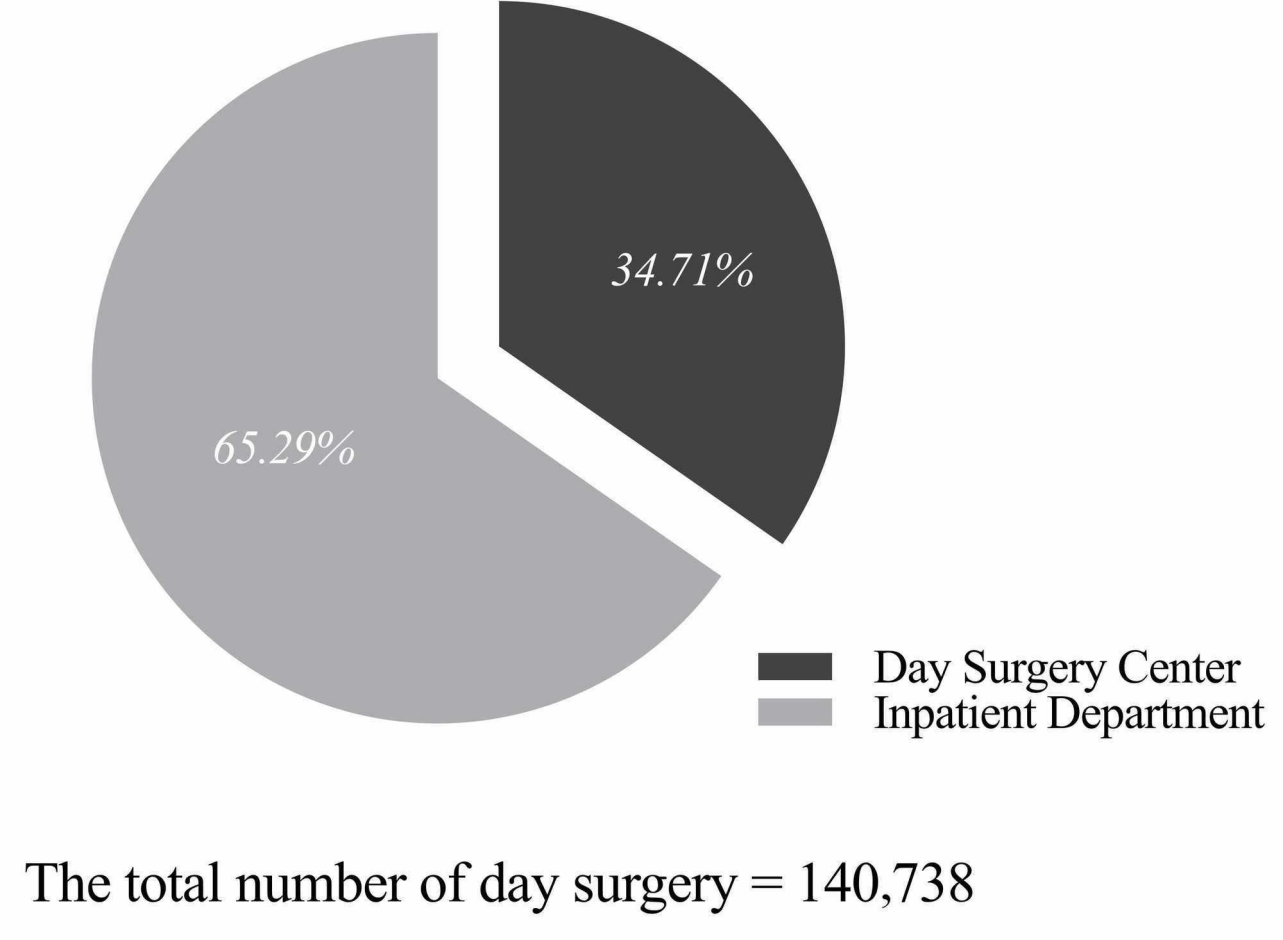
The distribution of total day surgery cases between the Day Surgery Center and Inpatient Departments at WCH from 2010 to 2018. WCH, West China Hospital

**Figure 4:**
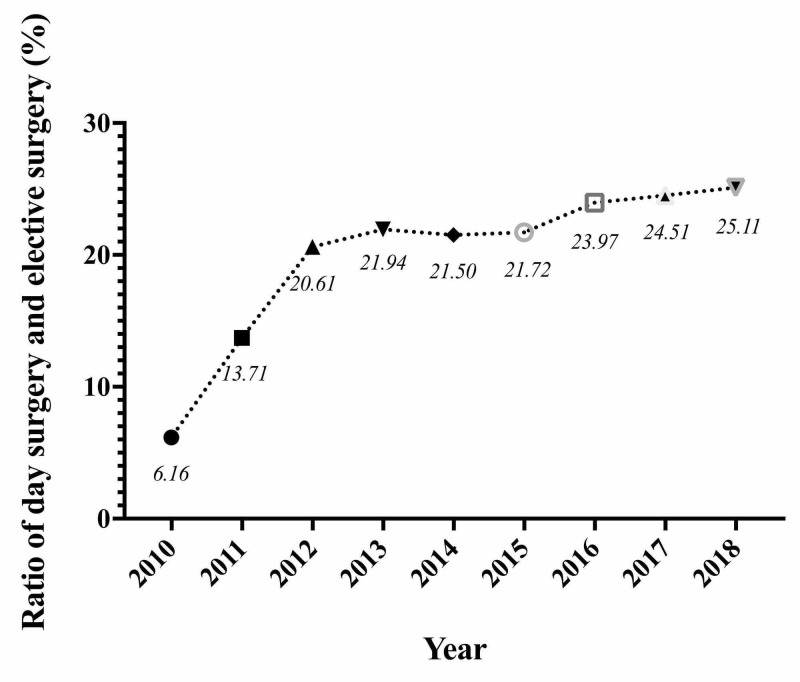
The increasing ratio of Day Surgery Center to inpatient surgery from 2010 to 2018 at West China Hospital.

From January 1, 2010, to December 31, 2018, WCH performed 140,738 day surgery cases, of which 48,854 (34.71%) cases were performed in the Day Surgery Center and 91,884 (65.29%) cases were performed in Inpatient Departments (Figures 2, 3). The ratio of day surgery to inpatient surgery has been on the rise from 6.16% in 2010 to 25.11% in 2018 (Figure 4). Inguinal hernia repair, LC, vein stripping and ligation, and excision of colonic polyps were performed in the Day Surgery Center. Table 1 shows several main surgery types in the Day Surgery Center and Inpatient Departments.

**Table 1:** Main day surgery types in central mode and mix mode at WCH from 2010 to 2018. LC, laparoscopic cholecystectomy; WCH, West China Hospital

Central Mode (Day Surgery Center)	Mix Mode (Inpatient Departments)
LC	Pediatric internal fixation removal
Inguinal hernia repair	
Excision of colonic polyps	Transurethral resection of bladder tumor (including on-demand biopsy)
Excision of gastric polyps	
Vein stripping and ligation	Removal of cystolith and calculus of lower urethra (including use of holmium laser)
Excision of breast masses	
Adenoidectomy	Circumcision
Pediatric laparoscopic hernia repair	Renal cyst decortication
Pediatric resection of hydroceles	
Pediatric phalloplasty	Pterygium excision
Pediatric orchidopexy	Infraorbital suture correction
Pediatric hypospadias repair	Orbital lesion resection

**Figure 5:**
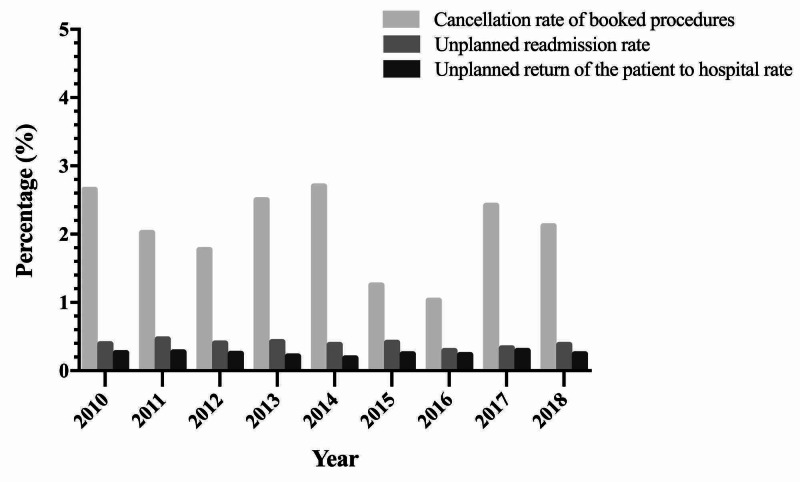
Cancellation rate of booked procedures, unplanned readmission rate, and unplanned return of the patient to hospital rate from 2010 to 2018.

**Figure 6:**
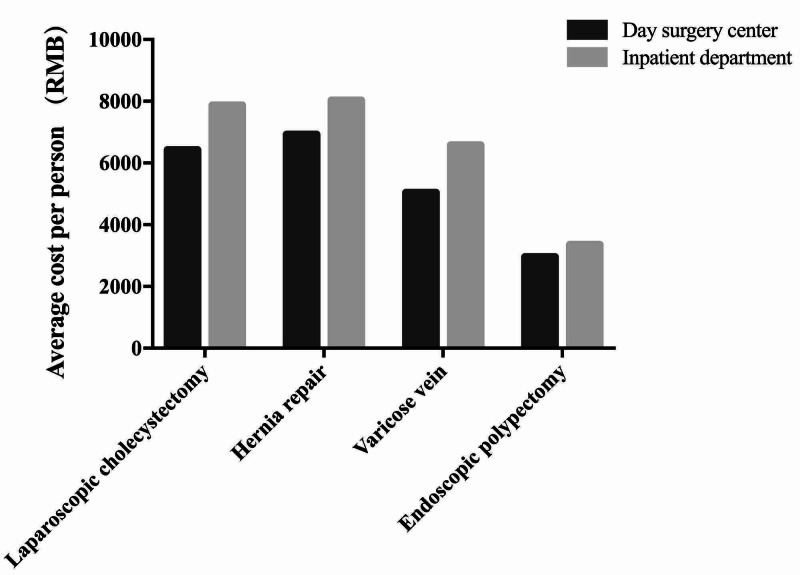
The average cost per person in the WCH Day Surgery Center versus Inpatient Departments for four common surgeries. WCH, West China Hospital

**Figure 7:**
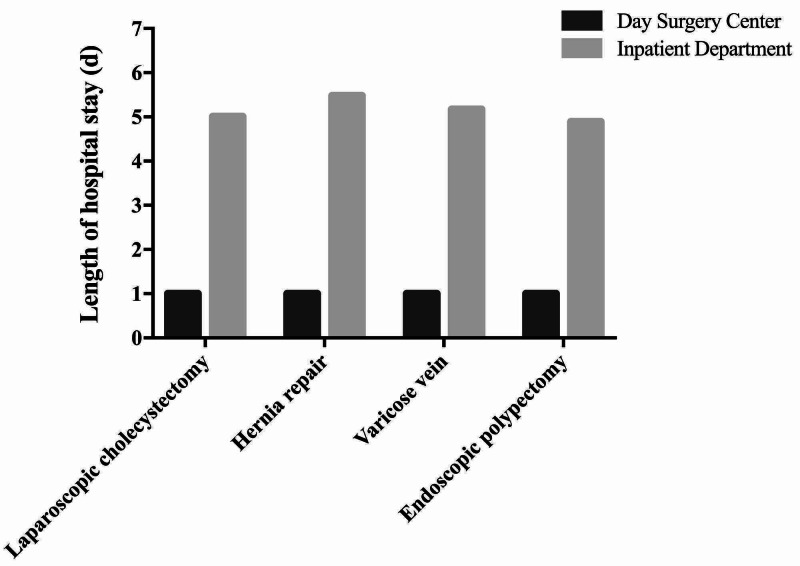
The average length of stay in the WCH Day Surgery Center and Inpatient Departments for four common surgeries. WCH, West China Hospital

As shown in Figure 5, the cancellation of booked procedures rate in Day Surgery Center is 2.65% in 2010 and 2.12% in 2018. The unplanned readmission rate and return to hospital rate are both stable at 0.40% and 0.25%, respectively. Unplanned readmission rate fluctuates around 0.25% from 2010 to 2018. The average cost for the most common surgeries, such as laparoscopic cholecystectomy, hernia repair, and endoscopic polypectomy, is between 3,000 to 6,000 RMB in Day Surgery Center. However, the costs for the same procedures completed in an Inpatient Department runs between 3,500 and 8,000 RMB, representing approximately 15% cost savings through the Day Surgery Center (Figure 6). The average length of stay for the most common procedures is 4.89-5.48 days when completed Inpatient Department (Figure 7) versus 24 hours for completion of the same procedures in the WCH Day Surgery Center.

## Discussion

The definition of day surgery varies from country to country and was influenced by local practices. In China, the concept of day surgery even varies from different healthcare institutions across the country. According to the literature, the Chinese Ambulatory Surgery Alliance (CASA) defines day surgery as a planned surgery that is performed, and the patient is discharged within 24 hours, excluding outpatient surgery. The maximum duration of hospitalization is less than 48 hours [23], but hospitals in Shanghai that carry out day surgery are in accordance with less than 48-hour discharge standard. Also, the vast majority of day surgery patients will have overnight stay in hospital, which is the difference compared with day surgery in the United States. As Figure 7 shows the average hospitalization time of day surgery in WCH is one day.

Currently, day surgery has dominant the surgery in some European countries and the United States, with around 65% of elective surgery being performed as day procedures in the United Kingdom and around 70% in the United States [24], due to its characteristics of “short and fast”, shorter waiting time and length of hospital stay, fast recovery based on enhanced recovery after surgery (ERAS) theory [25]. Thus, it reduces a large number of inpatient surgeries, we can tell from Figure 4, approximate 1/4 of all surgeries were performed as day surgery at WCH. China has issued a number of national policies that have been vigorously promoting day surgery since 2015 [26]. Since then, patients and health professionals have better knowledge of day surgery, which is quite beneficial for its development. With WCH being a leading teaching hospital in China and a general medical center in southwest, day surgery program can relieve the surgical treatment pressure in the hospital and southwest China area.

Due to the different domestic situations, only some of the clinical indicators recommended by the International Association for Ambulatory Surgery (IAAS) can be applied to day surgery in China [27]. We allow cancellation of booked procedures to provide evidence of the effectiveness of the booking system in Day Surgery Center, as well as unplanned return and readmission rates, to reflect possible problems in the performance of procedures. Unplanned return of the patient to the hospital and readmission rates are important indicators, but the data are hard to evaluate because they are difficult to differentiate or separate according to some studies [22,28]. There are no standards for all due to variations in each country. However, it has suggested that an acceptable readmission rate should between 1% and 2% [29]. As for return rates, it has a wide spectrum due to different studies and different surgeries [30]. Our study shows that unplanned readmission rate and return rate are both stable at 0.40% and 0.25% respectively; it is lower than the most of the reporting literature.

## Conclusions

Day surgery reduces the strain on the healthcare system in China resulting from increased medical demand. Day surgery in WCH is a safe and cost-effective way to meet the soaring medical demands in southwest China. Day surgery has its own advantages compared with traditional inpatient surgery. Furthermore, it cuts costs, shortens the length of stay, and allows the hospital to accommodate more patients.
